# Uncovering the Physiological Mechanisms Underlying the Roe Deer (*Capreolus capreolus*) Testicular Cycle: Analyses of Gelatinases and VEGF Patterns and Correlation with Testes Weight and Testosterone

**DOI:** 10.3390/ani10030444

**Published:** 2020-03-06

**Authors:** Alberto Elmi, Augusta Zannoni, Nadia Govoni, Martina Bertocchi, Monica Forni, Domenico Ventrella, Maria Laura Bacci

**Affiliations:** Department of Veterinary Medical Sciences, University of Bologna, via Tolara di Sopra 50, 40064 Ozzano dell’Emilia (BO), Italy; alberto.elmi2@unibo.it (A.E.); augusta.zannoni@unibo.it (A.Z.); nadia.govoni@unibo.it (N.G.); martina.bertocchi3@unibo.it (M.B.); monica.forni@unibo.it (M.F.); marialaura.bacci@unibo.it (M.L.B.)

**Keywords:** *Capreolus capreolus*, roe deer, gelatinases, proMMP2, TIMP, VEGF, testicular cycle, reproductive physiology, seasonal breeder

## Abstract

**Simple Summary:**

The roe deer (*Capreolus capreolus*) is a wild, small ruminant common in Europe and Asia with a peculiar reproductive cycle. The adult male shows a complete suspension of spermatozoa production during the winter that start again in spring with the peak of sexual activity in summer (July–August). The physiological mechanism underlying such a cycle is yet to be clarified. The work aimed at the analysis of some molecules, like gelatinases (MMP) and vascular growth factor, physiologically involved in tissue remodeling, in roe deer samples collected before (June–July) and after (August–September) the rutting period. Samples were provided by hunters of the Bologna Apennines area (Italy) according to the regional hunting plan. The result showed a post-rut reduction of testicular weight and testosterone, indicative of testes involution, correlated with an increase in pro-MMP2, the latent gelatinase capable of sustaining spermatogenesis once activated. It can be assumed that gelatinases are involved in the testicular cycle and start accumulating after the rutting period to be then activated in preparation of the next reproductive season inducing spermatozoa development and migration. Future studies on this pathway during all seasonal testicular cycles will provide more information about the interesting reproductive physiology of roe deer.

**Abstract:**

The roe deer (*Capreolus capreolus*) represents a spontaneous model of testicular inactivation: During winter, bucks show a suspension of spermatogenesis that starts again in spring and peaks during the breeding season (July–August). The underlying mechanisms to the regulation of the cyclic testicular changes are still not fully clear but seem to be imputable to the spermatogenic cell line since other testicular cell populations remain stable without apoptotic phenomena. The aim of the study was to investigate apoptosis, gelatinases (MMP2 and 9), their inhibiting factors (TIMP 1-2), and two isoforms of vascular endothelial growth factor (VEGF121 and 165) with its receptors (VEGFR1-2) in testes collected during pre- and post-rut periods, and to correlate them with testicular weight (TW) and testosterone (TEST). Testes from 18 adult sexually mature bucks were collected in Bologna Apennines (Italy). Samples were weighed and parenchyma collected. Radioimmunoassay, real-time PCR, and zymography were performed. The results showed a post-rut decrease in TW and TEST and an increase in proMMP2, also highlighting a correlation between the gelatinases and the testicular functionality. The VEGF pattern did not show modifications nor correlation with TW and TEST. Overall, gelatinases and their inhibitors, described herein for the first time in roe deer testes, seem to play an important role in the testicular cycle.

## 1. Introduction

In light of its peculiar reproductive physiology, the roe deer (*Capreolus capreolus*, Linnaeus 1758) represents a spontaneous model of seasonal regulation of testicular function [1]. Indeed, during winter, sexually mature males, called bucks, show a complete suspension in spermatogenesis. The cycle starts again in spring, with the highest peak of hormonal and reproductive activity recorded later on during the breeding season [2,3,4,5,6]. The reproductive season is conventionally divided into three periods: The pre-rut period (May–mid July), the short rutting period in sync with females’ oestrus (mid-July–mid-August), and the post-rut period (mid-August–September). Testicular involution, that already initiates at the end of the rutting period, is characterized by a drastic reduction in testicular weight (up to 20%) and a decline in testosterone levels, resulting in functional inactivation of the gonads [2,7,8,9]. The cyclic growth and involution of the testes depends on histo-morphological changes in cellular composition: The somatic components (Sertoli and Leydig cells) remain numerically constant despite functional and morphological modifications, while the germ cell line shows a sharp decline in mitotic/meiotic cell figures [10,11,12]. To the best of the authors’ knowledge, the underlying mechanisms to such cyclic annual testicular changes are still not fully clear and need to be further studied. To fill this gap, several growth factors were investigated including: Transforming growth factors (TGF-α, TGF-β1 and TGF-β3), fibroblast-like growth factors (FGF-1 and FGF-2), and insulin-like growth factors (IGF1 and IGF2) [13,14,15]. Each of the afore-mentioned factor showed a seasonal variability, more or less accentuated, and dependent on the different testicular cell types involved [4].

In other organs showing temporary functional cycles, such as the *corpora lutea*, a pivotal role in angiogenic/angioregression and tissue remodeling phenomena has been assigned to the vascular endothelial growth factor (VEFG) and gelatinases pathways and apoptotic processes [16,17]. It may therefore be hypothesized that such pathways are also involved in the roe deer testicular cycle. Nonetheless, based on the published literature, inhibition/activation of apoptotic phenomena does not seem to be involved in roe deer testicular involution [18]. The VEGF, upon binding to its tyrosine kinases specific receptors (VEGFR1 and VEGFR2), induces proliferation of endothelial cells, increases permeability of vessels’ walls, promotes cell migration, and inhibits apoptosis [19,20]. On the testes, VEGF seems to play a role in the modulation of blood–testicular barrier (BTB) permeability, in the regulation of germ cells differentiation and their migration, and in blood vessel development [20,21,22]. In roe deer testes, the highest levels of VEGF mRNA expression were observed during the pre-rut period, with subsequent reduction [23,24]. Testicular variation of VEGF expression were also reported in other seasonal species such as the white-footed mice [25] and the European bison [25]. Regardless of the timing of angiogenesis, the first step before growth factors recruitment and epithelial cells proliferation is the degradation of the epithelial layer of the existing vessel operated by the matrix metalloproteinases (MMPs) [26]. The latter play a role in the activation of the pro-angiogenic factors, exerting an important cross-talk with VEGF both in normal and pathological angiogenesis [27,28].

The MMP family is a group of calcium and zinc-dependent endoproteases expressed as zymogens (pro-MMPs), which are processed into active forms (act-MMPs) by enzymatic proteolysis [29]. Overall, they are involved in tissue remodeling by degradation of extracellular matrix (ECM), and are regulated by their specific endogenous tissue inhibitors (TIMPs) [30]. MMPs, and in particular gelatinases (MMP2 and MMP9), have already been described in the reproductive system of different species [16,29] and are correlated with BTB modulation, spermatogenesis, and fertility [31,32,33,34,35,36,37]. In an ovine model of prolonged testicular thermal shock, the levels of MMP2 within seminal plasma temporarily dropped in correlation to azoospermia [38]. Within the testis, both MMP2 and 9 are secreted as proenzymes by Sertoli cells and, once activated, facilitate the migration of spermatids from the basal membrane toward the lumen by increasing BTB permeability [39,40].

Based on the analogies with other temporal reproductive organs like the *corpora lutea*, the aim of the present study was to investigate the peculiar reproductive physiology of the male roe deer by analyzing both the VEGF and gelatinases pathways. MMP2 and 9, their inhibiting factors (TIMP 1-2), and two isoforms of VEGF (121-165) with its receptors (VEGFR1-2) were analyzed in testes collected during pre- and post-rut periods. To confirm the already described testicular morpho-functional involution, testicular testosterone and weight were also recorded and correlated to the afore-mentioned parameters.

## 2. Materials and Methods

### 2.1. Animals and Sampling

Eighteen sexually mature roe deer bucks (n = 18) were sampled during the 2018 hunting season in the South-Western Bologna Apennines (Italy) according to the regional hunting plan (Resolution No. 792/2018 of the Emilia Romagna Regional Executive). Half of the animals were hunted between June 1st and July 15th (pre-rut; n = 9), and the remaining half between August 15th and September 30th (post-rut; n = 9). Animals found dead within the territory during the hunting periods were not included in the study to avoid biases related to possible pathological conditions. Upon death, all animals were immediately transferred to the pertinent biometrical center where the personnel collected scrota, including testes and epididymis. Specimens were then moved within 2 hours at refrigerated temperature (5 ± 1 °C) to the physiology laboratories (ANFI-ASA) of the Department of Veterinary Medical Sciences of the University of Bologna (Ozzano dell’Emilia, Italy) [3]. Ages were assessed upon analyses of teeth eruption and wear patterns as previously described [41]. Since all the biological specimens analyzed in this study were obtained from hunted animals (in accordance with the hunting plan in force), no ethical approval was necessary.

### 2.2. Testicular Weight and Tissue Sampling (TW)

Testes were isolated from the scrota and weighed using a lab scale (FCB 12K1, KERN & SOHN GmbH, Balingen, Germany) after epididymis ablation. The weights of the two gonads (TW) were averaged. Testes were cut in half and parenchyma from the central area was collected, minced with a razor blade, and divided into 2 aliquots: One immediately flash frozen in liquid nitrogen and stored at −80 °C (for MMPs and testosterone quantification and oligonucleosomes detection), the other one stored in RNA Stabilization Solution (RNAlater™, Thermo Fisher Scientific, Waltham, MA, USA) for 24 h at +4 °C and then moved at −80 °C after solution removal (for RNA extraction).

### 2.3. DNA Isolation for Oligonucleosomes Detection

Low-molecular-weight DNA was isolated from testes as previously described [17]. As positive control, a porcine regressing *corpus luteus* sample was used. DNA samples (50 µg/lane) were separated according to size by electrophoresis in a standard 2% agarose gel stained with ethidium bromide (EtBr) and visualized under ultraviolet light. Gel images were captured with a computerized system (Chemidoc Instrument, Bio-Rad, Hercules, CA, USA).

### 2.4. Testicular Testosterone Analysis by RIA (TEST)

Tissue samples were homogenized for 1 min in PBS at a final concentration of 100 mg/mL to prepare 10% (w/v) homogenate, centrifuged at 3000× *g* for 10 min to sediment the insoluble debris; the supernatants were stored at −80 °C until analysis. Steroids were extracted overnight by mixing 0.2 mL of homogenate with 5 mL of methanol on a rotary mixer. After centrifugation (2000× *g* for 10 min), 4 mL of the methanol phase were collected, transferred into a glass tube and evaporated to dryness under an air-stream suction hood. The dried extracts were then stored at −20 °C until reconstitution in assay buffer (1 mL) and 0.05mL (0.8 mg tissue equivalent) was used for testosterone (TEST) quantification by radioimmunoassay; tritiated TEST (30 pg/tube; 83.4 Ci/mmol; PerkinElmer inc. Boston, MA, USA) was added, followed by rabbit anti-testosterone serum (0.1mL, 1:50,000) produced in our laboratory. After incubation and separation of antibody-bound and –unbound steroid by charcoal-dextran solution (charcoal 0.25%, dextran 0.02% in phosphate buffer), tubes were centrifuged (15 min, 3000× *g*), the supernatant was decanted, and radioactivity immediately measured using a β-scintillation counter (Packard C1600, Perkin Elmer, Waltham, MA, USA). The sensitivity of the assay was 4.05 pg/tube, the precision within test was assessed by calculating intra-assay coefficients of variation from all duplicated samples analyzed and was 5.7%. Cross reactions of various steroids with antiserum raised against testosterone were testosterone (100%), dihydrotestosterone (25.44%), androstenedione (0.6%), 17β-estradiol, progesterone, and cortisol (<0.0001%).

In order to determine the parallelism between hormone standards and endogenous hormone in testicular tissue of the roe deer, a pooled sample containing high concentrations in TEST was serially diluted (1:1–1:8) with assay buffer. A regression analysis was used to determine parallelism between the two hormone levels in the same assay. A high degree of parallelism was confirmed by regression test (r2 = 0.99). To determine the recovery of the extraction, five samples were spiked with a small amount (~2000 cpm) of [^3^H] testosterone and the percentage of radioactivity recovered (96.5%) was calculated. The assay results were expressed as pg/mg of tissue.

### 2.5. RNA Extraction and Real-Time qPCR for VEGF121, VEGF165, VEGFR1, VEGFR2, TIMP1 and TIMP2

Total RNA extraction was performed using TRI Reagent (Molecular Research Center In, Cincinnati, OH, USA) and NucleoSpin RNA II (Macherey-Nagel GmbH & Co. KG, Düren, Germany) kit according to the manufacturer’s instructions. Tissue was homogenized in TRI Reagent (30 mg/ml) with an Ultra Turrax, then 200 µL of chloroform were added to the suspension and mixed well. After incubation at room temperature (10 min), samples were centrifuged (12,000× *g* for 10 min) and the aqueous phase recovered. An equal volume of absolute ethanol (99%) was added and the resulting solution was applied to the NucleoSpin RNA Column. After spectrophotometric quantification, total RNA (250 ng) was reverse transcribed to cDNA using the iScript cDNA Synthesis Kit (Bio-Rad Laboratories Inc., Hercules, CA, USA) in a final volume of 20 μL. To evaluate gene expression profiles, quantitative real-time PCR (qPCR) was carried out in CFX96 thermal cycler (Bio-Rad) using SYBR green detection for target genes. 

Out of the target genes, sequences for VEGF121, VEGF165, VEGFR1, and VEGFR2 were based on roe deer (Table 1), while the ones for TIMP1 and TIMP2 on *Bos taurus* (QIAGEN, Hilden, Germany, RT2 qPCR Primer Assay for TIMP1 and TIMP2 Cat. No. PPB00865A, PPB00864A, respectively). Regarding the reference genes, glyceraldehyde-3-phosphate dehydrogenase (GAPDH) was based on roe deer sequence (Table 1), while hypoxanthine phosphoribosyltransferase 1 (HPRT1), beta-actin (ACTb), and beta-2-microglobulin (B2M) were based on *Bos taurus* sequences (QIAGEN, Hilden, Germany, RT2 qPCR Primer Assay for HPRT1, ACTb, B2M; Cat. No. PPB00330A, PPB00173A, PPS00031A, respectively). Specific primers for roe deer were designed using Beacon Designer 2.07 (Premier Biosoft International, Palo Alto, CA, USA).

All amplification reactions were performed in 20 μL and analyzed in duplicates; the reaction contained: 10 μL of iTaq Universal SYBR Green Supermix (Bio-RAD), 0.8 μL of forward and reverse primers (5 μM each) of each target gene, 2 μL cDNA, and 7.2 μL of water. The real-time program included an initial denaturation period of 1.5 min at 95 °C, 40 cycles at 95 °C for 15 s, and 60 °C for 30 s, followed by a melting step with ramping from 55 °C to 95 °C at a rate of 0.5b°C/10 s. To validate the primers chosen according to *Bos taurus* sequences, extraction and qPCR from a bovine testis were also performed. The specificity of the amplified PCR products was confirmed by agarose gel electrophoresis and melting curve analysis. The relative expressions of the studied genes were normalized based on the geometric mean of the three reference genes.

The relative mRNA expression of tested genes was evaluated using the 2-∆∆Ct method (fold changes) [42], in relation to pre-rut group, in which ∆Ct = Ct interest gene – Ct mean reference genes, and ∆∆Ct = ∆Ct pre-rut group − ∆Ct post-rut group.

### 2.6. MMPs Activity Assay

A portion of testis was homogenized in PBS (0.1 g/mL) by an Ultra Turrax. The obtained homogenate was processed as follows: 500 µl were centrifuged at 2000× *g* for 10 min at 4 °C and supernatant was stored at −20 °C until MMPs activity evaluation. MMP2 and MMP9 activities were analyzed by means of gelatin zymography on 10% Tris-Glycine poliacrylamide pre-cast gels with 0.1% gelatin (10% Novex Zymogram Plus Gels, Thermo Fisher Scientific, Rockford, IL, USA). Protein content of samples was determined by a Protein Assay Kit (TP0300, Sigma-Aldrich, St. Louis, MO, USA) following the manufacturer’s instructions. Five mL of each sample were mixed with an equal volume of sample buffer (Tris-Glycine SDS Sample Buffer 2X, Thermo Fisher Scientific) and loaded into the gel. Electrophoresis was performed with 1X Tris-Glycine SDS Running Buffer (Thermo Fisher Scientific) at a constant voltage (125 V for 90 minutes). Following electrophoresis, gels were washed for 30 min in 1X Zymogram Renaturing Buffer (Invitrogen, Renfrew, U.K.), equilibrated at room temperature for 30 min in developing buffer (1X Zymogram Developing Buffer, Thermo Fisher Scientific), and then incubated at 37 °C for 22–24 h in fresh developing buffer. Band of gelatinolytic activity were developed, after staining gels for 1 h with SimplyBlue™ Safestain (Thermo Fisher Scientific) and two washes in water. MMP2 and MMP9 bands were identified by comparison with a standard sample (porcine corpus luteum 17 days after ovulation) as previously reported [16]. Each analysis was repeated three times and the results were averaged. Gel images were captured with a computerized system Chemidoc Instrument (Bio-Rad) and gelatinolytic active area were measured with densitometric analysis software using Image Lab Software (Bio-Rad). The relative abundance of MMPs (mean ± SD) were expressed as arbitrary units (AU) in relation to the protein content (MMPs/protein).

### 2.7. Statistical Analyses

Statistical analyses were performed using the software R 3.0.3 (The R Foundation for Statistical Computing) and Prism and graphically represented using the software GraphPad Prism v.8 (GraphPad Software Inc., San Diego, CA, USA). The statistical significance for all tests was set at *p* < 0.05 (95% CI). Descriptive statistics were calculated and reported as mean, standard deviation (SD), and min/max values. Normal distribution of data was tested by means of the Shapiro–Wilk test. To assess the equality of variances in the two groups the Levene test was performed. Depending on the result of the previous assessments, parametric (Student or Welch *t* tests) or non-parametric tests (Mann–Whitney *U* tests) were applied to compare the groups. In order to evaluate the relationships between the different analyzed parameters, non-parametric Spearman correlation rank tests were performed (ρ > 0.50).

## 3. Results

The animals sampled in the present study were all sexually mature adults, with age ranging from 15 to 72 months, mean weight of 22.7 kg, and mean height at the withers of 75.3 cm. The descriptive results are summarized in Table S1.

### 3.1. Testicular Involution and Apoptosis

**Figure 1:**
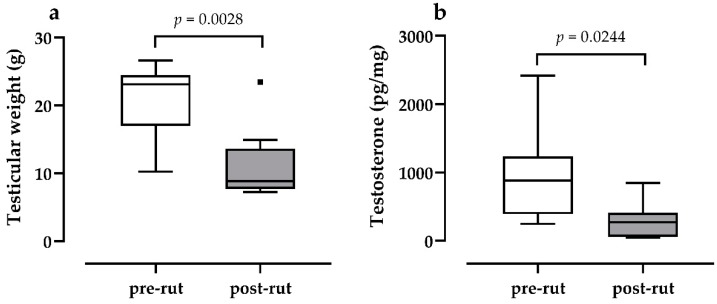
Differences between pre and post rut groups for (**a**) testicular weight (statistical analysis: Mann–Whitney *U* test) and (**b**) testicular testosterone (statistical analysis: Welch *t* test). ▪ = outlier.

The differences between pre- and post-rut periods for TW (Figure 1a) and TEST (Figure 1b) are reported in Figure 1. Both parameters show a statistically significant decrease in the post-rut period (TW *p* = 0.0028; TEST *p* = 0.0244).

DNA fragmentation was not detectable in both pre- and post-rut samples (data not shown).

### 3.2. Quantification of Tissue Expression of the Genes of Interest 

The specificity of all PCR products was verified in relation to melting curve analysis and agarose gel electrophoresis and in relation with testis from bovine when request. All the analyzed transcripts were detectable in all samples. 

**Figure 2:**
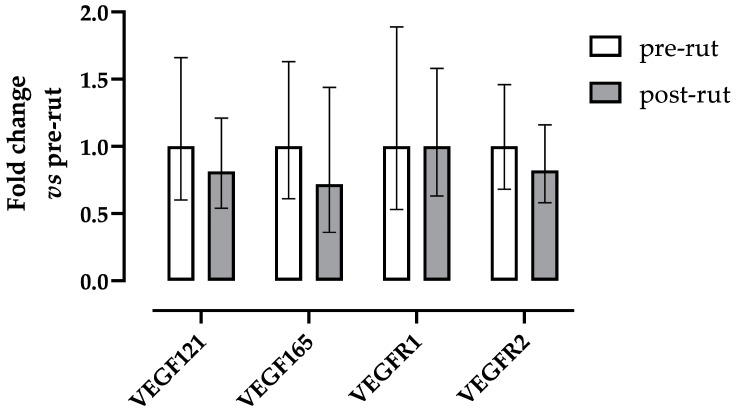
Relative gene expression of VEGF isoforms and their receptors in roe deer testes calculated as fold change in respect to the pre rut. Error bars represent the range of relative gene expression. No statistically significant differences were observed (VEGF121, VEGF165, and VEGFR2 were analyzed by means of Student *t* test: *p* = 0.6589, *p* = 0.4510, *p* = 0.5557 respectively; VEGFR1 was analyzed by means of Mann–Whitney *U* test: *p* = 0.3865).

**Figure 3:**
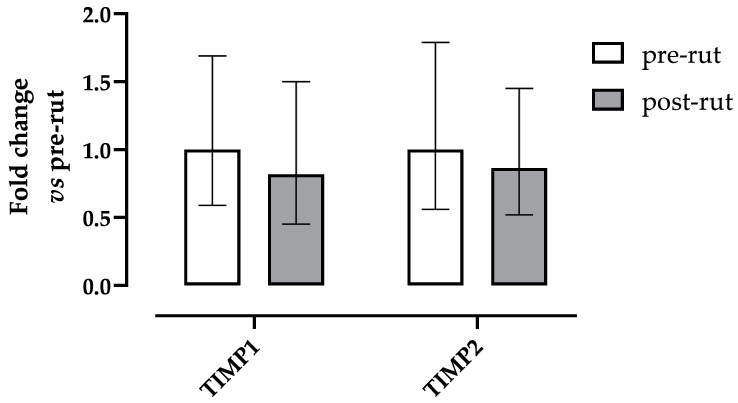
Relative gene expression of TIMP1 and TIMP2 in roe deer testes calculated as fold change in respect to the pre-rut. Error bars represent the range of relative gene expression. No statistically significant differences were observed (Student *t* test: TIMP1 *p* = 0.1372, TIMP2 *p* = 0.5894).

The expression levels of the two VEGF isoforms and its receptors did not show any significant difference between groups (Figure 2). Similarly, no significant differences were observed between groups for TIMP1 and TIMP2 expressions (Figure 3).

### 3.3. MMPs Activity

**Figure 4:**
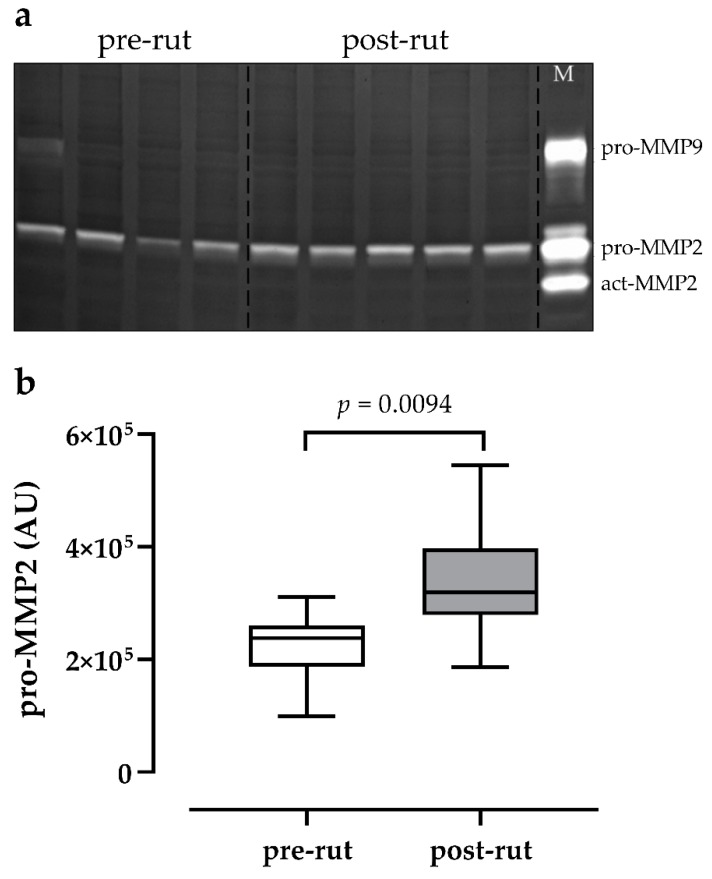
(**a**) Representative zymography gel showing gelatinase activity in pre (n = 9) and post-rut (n = 9) testis of Roe Deer. In the first lane, a l pro-MMP9 band was observed. Lane M = marker. (**b**) Relative abundance of latent pro-MMP2, expressed as arbitrary units (AU) on the basis of the protein content (means ± SEM). A statistically significant increase of gelatinase activity was observed in post-rut sample (Student *t* test).

Three distinct bands of gelatinase activity, corresponding to pro-MMP9, pro-MMP2, and active MMP2 (act-MMP2) were evidenced in the standard sample (Figure 4a, lane M).

In testis samples, the gelatinolytic activity of pro-MMP2 was observed in all samples, while no bands related to act-MMP2 were evidenced (Figure 4a). Only one pre-rut sample showed a weak band of pro-MMP9 (Figure 4a, first lane).

The activity of pro-MMP2 increased in the post-rut group as confirmed by the statistical analysis (*p* = 0.0094) (Figure 4b). 

### 3.4. Spearman Correlation Rank Analyses 

**Figure 5:**
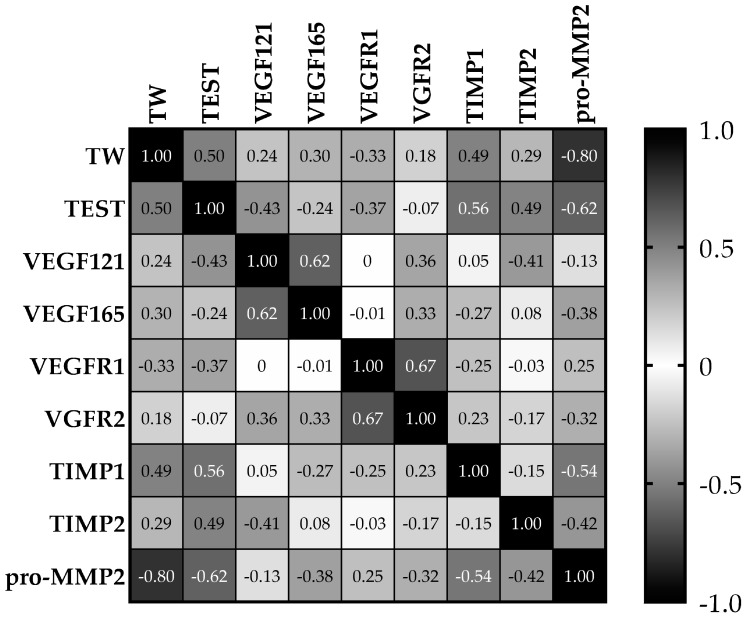
Colour-coded Spearman rank correlation coefficients (ρ) table. Black squares indicate perfect correlations (ρ = 1 or −1 in case of perfect inverse correlation); white squares indicate that the two variables do not vary together (ρ = 0); gray gradients indicate different levels of correlation.

The correlation coefficients (ρ) between the analyzed parameters are reported in Figure 5.

While ρ quantifies the direction and magnitude of correlation, a *p* < 0.05 rejects the idea that the correlation is due to random sampling. TW and TEST were correlated to each other (ρ = 0.50, *p* = 0.0353). Pro-MMP2 was correlated with TW (ρ = −0.80, *p* = 0.0001) and TEST (ρ = −0.62, *p* = 0.0058). The same situation was found for TIMP1 (TW ρ = 0.49, *p* = 0.0383; TEST ρ = 0.56, *p* = 0.0148), that also correlated with pro-MMP2 (ρ = −0.54, *p* = 0.0201). The other inhibitor, TIMP2, was only correlated with TEST (ρ = 0.49, *p* = 0.0399). Additionally, as expected, the two VEGF isoforms were correlated with each other (ρ = 0.62, *p* = 0.0058), like the two VEGF receptors (ρ = 0.67, *p* = 0.0025).

## 4. Discussion

The testicular cycle of the male roe deer (*Capreolus capreolus*) is characterized by a post-rut involution described by literature as a morphological reduction [7,11] and a decrease in hormonal levels evaluated on different matrices [4,6,10] including hair [9]. Data regarding TW and TEST reported in the present study perfectly align with the above-mentioned references, with a statistically significant decrease in post-rut samples when compared to pre-rut ones. Despite the correlation between testicular levels of testosterone and TW being significant (ρ = 0.50), it was less marked in respect to the previously reported correlation between plasmatic levels of the same hormone and TW (ρ = 0.76 [9]). Nonetheless, as expected, the results further confirm the already acknowledged post-rut morpho-functional testicular involution. Moreover, these data seem to provide more strength to the results despite the relative low sample size by confirming that the two sampling windows, dictated by the hunting calendar, were relevant.

Therefore, in order to try and better understand the mechanisms underlying the testicular post-rut involution and overall cycle, different angiogenic and tissue remodeling patterns were investigated. Pathways to be analyzed were chosen based on what is already known for other temporary functional reproductive tissues like the *corpus luteus*. This structure shows a peculiar morpho-functional cycle imputable to angiogenesis/angioregression phenomena, programmed cellular death (apoptosis), and, generally speaking, to tissue remodeling. All the latter cooperate to make the *corpus luteus* a transient endocrine gland, capable of producing hormones only when needed [16,17].

The vascular endothelial growth factor (VEGF) and its receptors are expressed in all testicular cells, both germ line and interstitial components [21], and play a pivotal role in male germ cell differentiation, proliferation, and migration [43,44]. The function of the VEGF/tyrosine receptor interaction within testicular physiology was studied in numerous species [20,25,45,46], including the roe deer [13]. The results of the present study did not show any statistical difference between pre- and post-rut periods in the gene expression of VEGF (isoforms 121 and 165) and its receptors (R1 and R2), confirming what was already reported by literature [23,24]. Despite the absence of statistical relevance, all analyzed parameters, an exception made for VEGFR1, showed a decreasing trend in the post-rut group. This may potentially be related to the already described VEGF down regulation during the rutting period [23,24]. No correlations were highlighted between the VEGF pattern (both the isoforms and the receptors) and TEST/TW, suggesting a lack of direct implication in the seasonal modulation of roe deer testicles. A wider sampling, potentially extended throughout the entire year, would help obtaining more in-depth information about the role of VEGF/receptors.

Apoptosis was not present in the analyzed samples, confirming what was already reported by a previous study [18]. Therefore, based on the results of both VEGF and this parameter, this study confirms that the structural modifications occurring in the roe deer testes are imputable only to the testicular germ line cells, thus to spermatogenesis suspension, and that no vascular remodeling is present.

Zymography was able to highlight the presence of pro-MMP2 in all analyzed samples and only a weak signal of pro-MMP9 in a single case, while TIMPs, investigated by means of qRT-PCR, were always present. No active forms of MMPs were detected. Such absence of active MMPs active forms and the lack of statistical differences in TIMPs expression are in agreement with the VEGF and apoptosis findings and indicate that the testicular involution in the post-rut period, confirmed by TW and TEST, is not related to overall remodelling processes. Nonetheless, the level of pro-MMP2, the latent form of MMP2, statistically increased in the post-rut group and, on the contrary, both TIMPs showed a decreasing trend. In such scenario, the role of the testicular germ line cells in the post-rut period seems to become clearer. Indeed, it has to be acknowledged that MMPs activity is not only related to tissue remodelling in general, but also, in the testes, to alterations in BTB permeability, functional development of seminiferous tubules, and the migration of immature forms of spermatozoa as previously reported [47]. What can be hypothesized is that the increase in the latent form of MMP2 after the rutting period may indicate the reactivation of Sertoli cells, responsible for gelatinases production, in preparation of the next spermatogenic cycle. The accumulation of pro-MMP2 during the post-rut period will be most likely followed by its activation later on during the year, in late winter, or early spring, when such spermatogenic phenomena take place in this species [10]. Another important aspect worth mentioning is the fact that, in other mammals like dogs, high levels of pro-MMP2 have been associated with poor semen quality [48], matching what happens in post-rut bucks [49]. In such a scenario, the highlighted correlations between MMP-related analytes and testicular involution should be imputable to the suspension in spermatogenesis in the post-rut period. To the best of the author’s knowledge, this work represents the first report regarding gelatinases and their specific inhibitors in the roe deer testes, therefore discussions can be challenging.

On the basis of the results, when looking at the comparison with the *corpus luteus*, the roe deer testes seems to have different biological mechanisms and pathways contributing to its peculiar cycle. Indeed, apoptosis and vascular remodelling are not involved, at least during pre- and immediate post-rut periods, and gelatinases seem to contribute by modulating different processes. In order to have a clearer vision, extended samplings covering the entire year are needed and would help uncovering the correlations between the gelatinases’ pathway and the testicular cycle.

Finally, the present study confirms the already proposed possibility to collaborate with biometrical centres in order to collect samples from hunted animals [9,50]. Such opportunistic sampling allows to gain knowledge regarding the physiology of wild animals, relatively undisturbed by human influences.

## 5. Conclusions

In conclusion, the present work provides new data regarding the peculiar male reproductive physiology of the roe deer (*Capreolus capreolus*), confirming the morpho-functional reduction of the testes in the post-rut period. Despite the lack of correlation of the analyzed VEGF pattern parameters with testicular weight and testosterone, the results are aligned with previous reports. Gelatinases and their inhibitors, described herein for the first time in roe deer testes, seem to play an important role in such peculiar cycle, most likely upon modulation of spermatogonial line development and migration. Being able to extend the sampling to the entire year would help clarifying the role of such patterns in the seasonal testicular cycle with more accurate timing, and potentially provide information translatable to other species.

## Figures and Tables

**Table 1:** Specific roe deer primer sequences used for RT-qPCR.

Gene		Primer Sequence (5′-->3′)	PCR size (bp)	Accession Number	Reference
VEGF121	For:	GTTCATCTTCAAGCCGTCCTGTG	130	AF 152593	Present study
	Rev:	TTGGTGAGGTTTGATCCGCATAATC			
VEGF165	For:	CCACCGAGGAGTTCAACATCAC	177	AF 152594	Present study
	Rev:	CAAACAAATGCTTTCTCCGCTCTG			
VEGFR1	For:	GAGTCACGGAAGAGGATG	171	NM_001191132	Present study
	Rev:	TTAACAGGAGCCAGAAGAG			
VEGFR2	For:	GGCTACTTCTTGTCATCGTTCTAC	137	NM_001110000	Present study
	Rev:	TCGTAAGGCAGGCGTTCAC			
GAPDH	For:	CACCGTCCATGCCATCAC	109	AF363637	Present study
	Rev:	CTCCGATGCCTGCTTCACTACCTT			

VEGF121: Vascular endothelial growth factor isoform 121; VEGF165: Vascular endothelial growth factor isoform 165; VEGFR1: Vascular endothelial growth factor receptor 1; VEGFR2: Vascular endothelial growth factor receptor 2; GAPDH: glyceraldehyde-3-phosphate dehydrogenase; bp: base pair; for: forward; rev: reverse.

## Supplementary Materials

The following are available online at https://www.mdpi.com/2076-2615/10/3/444/s1, Table S1: Descriptive statistics for the analyzed parameters divided per group.
