# Supplementary material for: Uncovering the Physiological Mechanisms Underlying the Roe Deer (Capreolus capreolus) Testicular Cycle: Analyses of Gelatinases and VEGF Patterns and Correlation with Testes Weight and Testosterone
Source: Animals (Basel). 2020 Mar 6;10(3):444. doi: 10.3390/ani10030444 (PMC7143327; doi:10.3390/ani10030444)
Supplement: Supplementary file 1 [file animals-10-00444-s001.pdf]

Supplementary file

Uncovering the physiological mechanisms underlying the roe deer (*Capreolus capreolus*) testicular cycle: analyses of gelatinases and VEGF patterns and correlation with testes weight and testosterone

Alberto Elmi, Augusta Zannoni, Nadia Govoni, Martina Bertocchi, Monica Forni, Domenico Ventrella and Maria Laura Bacci

Table S1: Descriptive statistics for the roe deer data divided per group.

	Pre-rut			Post-rut		
	**mean (SD)**	**min ; max**	**normal distribution *p* value**	**mean (SD)**	**min; max**	**normal distribution *p* value**
**Age** months	39 (15)	24; 72	0.037	32 (10)	15; 48	0.744
**Body Weight** kg	23.9 (6.0)	17.5; 32.5	0.788	22.0 (2.2)	19.0; 26.0	0.808
**Height at the withers** cm	74.58 (3.17)	69.30; 77.50	0.254	75.62 (2.99)	70.20; 78.00	0.021
**Testicular Weight** g	20.47 (5.26)	10.24; 26.61	0.358	11.11 (5.26)	7.20; 23.42	0.006
**Testosterone** pg/mg	917.39 (676.30)	248.87; 2419.75	0.121	281.18 (261.18)	48.22; 848.00	0.069
**pro–MMP2** AUx10^5	2.24 (0.61)	9.84; 3.11	0.484	3.41 (1.01)	1.86; 5.45	0.806
**VEGF 121** ΔCT	−2.40 (0.68)	−3.28; −1.33	0.551	−2.53 (0.58)	−3.46; −1.49	0.948
**VEGF 165** ΔCT	−4.07 (0.63)	−4.75; −2.99	0.162	−4.38 (1.01)	−6.29; −2.73	0.885
**VEGFR1** ΔCT	−5.08 (0.96)	−6.70; −3.08	0.335	−4.79 (0.66)	−5.69; −3.97	0.019
**VGFR2** ΔCT	−2.80 (0.57)	−3.79; −1.80	0.899	−2.95 (0.50)	−3.50; −1.86	0.218
**TIMP1** ΔCT	−0.90 (0.79)	−2.05; 0.09	0.398	−1.51 (0.88)	−2.74; −0.17	0.458
**TIMP2** ΔCT	1.63 (0.74)	0.58; 2.50	0.105	1.44 (0.74)	0.19; 2.28	0.318

Normal distribution was assessed by means of the Shapiro–Wilk test (C.I. 95%).
